# Application of Artificial Intelligence in Geriatric Care: Bibliometric Analysis

**DOI:** 10.2196/46014

**Published:** 2023-06-23

**Authors:** Jingjing Wang, Yiqing Liang, Songmei Cao, Peixuan Cai, Yimeng Fan

**Affiliations:** 1 Department of Nursing The Affiliated Hospital of Jiangsu University Zhenjiang China; 2 Medical College Jiangsu University Zhenjiang China; 3 Department of Geriatrics The Affiliated Huaian No 1 People's Hospital of Nanjing Medical University Huaian China

**Keywords:** artificial intelligence, older adults, geriatric care, bibliometric analysis

## Abstract

**Background:**

Artificial intelligence (AI) can improve the health and well-being of older adults and has the potential to assist and improve nursing care. In recent years, research in this area has been increasing. Therefore, it is necessary to understand the status of development and main research hotspots and identify the main contributors and their relationships in the application of AI in geriatric care via bibliometric analysis.

**Objective:**

Using bibliometric analysis, this study aims to examine the current research hotspots and collaborative networks in the application of AI in geriatric care over the past 23 years.

**Methods:**

The Web of Science Core Collection database was used as a source. All publications from inception to August 2022 were downloaded. The external characteristics of the publications were summarized through HistCite and the Web of Science. Keywords and collaborative networks were analyzed using VOSviewers and Citespace.

**Results:**

We obtained a total of 230 publications. The works originated in 499 institutions in 39 countries, were published in 124 journals, and were written by 1216 authors. Publications increased sharply from 2014 to 2022, accounting for 90.87% (209/230) of all publications. The United States and the International Journal of Social Robotics had the highest number of publications on this topic. The 1216 authors were divided into 5 main clusters. Among the 230 publications, 4 clusters were modeled, including Alzheimer disease, aged care, acceptance, and the surveillance and treatment of diseases. Machine learning, deep learning, and rehabilitation had also become recent research hotspots.

**Conclusions:**

Research on the application of AI in geriatric care has developed rapidly. The development of research and cooperation among countries/regions and institutions are limited. In the future, strengthening the cooperation and communication between different countries/regions and institutions may further drive this field’s development. This study provides researchers with the information necessary to understand the current state, collaborative networks, and main research hotspots of the field. In addition, our results suggest a series of recommendations for future research.

## Introduction

The older adult population is growing rapidly and posing unprecedented challenges worldwide. The United Nations (UN) data shows that the older adult population aged ≥60 years is expected to reach around 2 billion worldwide by 2050 [1]. The rapid growth of aging is putting enormous pressure on some societies and countries, impacting their economies, workforce structures, and social security systems [2]. As adults age, their physical function declines and they are at high risk for chronic diseases. Fong [3] showed that 85% of older people have at least 1 chronic illness. The demand for medical services for older people is increasing [4]. In addition, many countries are facing a shortage of caregivers, with a global shortage of 17 million health care providers in 2019 [5,6].

Artificial intelligence (AI) is a science that involves multiple fields of knowledge and encompasses a wide range of techniques and aims to simulate and extend human intelligence through machines, and research fields include expert systems, machine learning, robotics, decision support systems, and pattern recognition [7,8]. AI is developing quickly [8]. It has already been used in geriatric care, alleviating the shortage of caregivers and the uneven distribution of resources [6,9]. A growing number of researchers believe that AI can effectively address the unmet needs of older adults further by reallocating the distribution of nurses and other health care resources. Many researchers have conducted studies on the application of AI in geriatric care, such as the care for patients with Alzheimer disease, geriatric care, disease recognition, and medication reminders [10-16].

Nevertheless, the field’s research themes and trends are not yet clear. Currently, there are no studies that analyze the current status and hotspots of research on the application of AI in geriatric care in the form of bibliometrics.

Bibliometrics refers to the quantitative synthesis of publications using mathematical and statistical methods. This method analyzes the development trends, distribution structure, and relationships within a specific field based on the external characteristics of publications, such as the number of publications, authors, and journals. In this study, we used bibliometric methods to conduct a comprehensive analysis of the research related to the application of AI in geriatric care in order to summarize the status of research in the field. This research also analyzed research hotspots and development trends as a reference for future research directions and researchers.

## Methods

### Data Sources and Search Strategy

We retrieved the publications used in this study from the Web of Science Core Collection from inception to August 2022. We formulated a search strategy concurrent to reading the relevant literature that had been identified previously. Our search used the following keywords: (“artificial intelligence” OR “machine intelligence” OR “robot*” OR “robot technology” OR “assistant robot” OR “robot-assisted” OR “computational intelligence” OR “computer reasoning” OR “deep learning” OR “computer vision system” OR “sentiment analysis” OR “machine learning“ OR “neural network*” OR “data learning“ OR “expert* system*” OR “natural language processing“ OR “support vector machine*” OR “decision tree*” OR “data mining“ OR “deep learning” OR “neural network*” OR “bayesian network*” OR “intelligent learning” OR “feature* learning” OR “feature* extraction” OR “feature* mining” OR “feature* selection” OR “unsupervised clustering” OR “image* segmentation“ OR “supervised learning” OR “semantic segmentation” OR “deep network*” OR “neural learning” OR “neural nets model“ OR “graph mining“ OR “data clustering“ OR “big data“ OR “knowledge graph”) AND (“senior∗” OR “aged∗” OR “elder∗” OR “geri∗” OR “age∗” OR “older adult∗” OR “geriatric∗” OR “aging” OR “very elderly” OR “frail elderly”) AND (“nurs*” OR “care” OR “psychological care” OR “bibliotherapy”). Inclusion criteria were limited to studies that (1) involved AI technologies, (2) were related to older adults, and (3) were published as papers. Exclusion criteria were also defined with source types, such as conference papers, reviews, data papers, editorial materials, and retraction. After the first search, all the literature was screened and checked separately by 2 researchers to ensure that all the papers used were relevant to the study topic. In the case of a dispute, we consulted a third investigator.

### Analysis Tools

The following tools were used to analyze the literature:

- HistCite 12.03.17, the history of citation, is a citation analysis tool developed by Eugene Garfield, the inventor of the Science Citation Index (SCI). HistCite can provide an overview of the history of a field’s development and recognize the most influential literature and authors [17]. It was used in this study to generate all tables.
- VOSviewer 1.6.18, a free Java software for document mapping, was developed by the Centre for Science and Technology Studies, University of Leiden, the Netherlands. It was used in this study to analyze collaborative networks and the keyword co-occurrence network.
- We used the Bibliometrix Online Analysis Platform to present the collaborative networks among countries/regions [18,19]. It was used in this study to analyze a network map of scientific cooperation among countries/regions.
- Citespace 6.2.R2, a scientometric tool developed by Chaomei Chen, was used to report burst keywords in this study [20].

### Data Analysis

The analysis function in the Web of Science database was used to summarize external characteristics, including the publication number and the average number of citations. Literature records and cited reference data were downloaded in text form. Keywords with similar meanings but different spellings or expressions were merged (eg, “elder people” and “older adults”). Furthermore, general and nonsensical keywords were removed (eg, “men,” “scale”). Once cleaned, the data were imported into the aforementioned software separately for internal structure analysis.

### Ethical Considerations

The data used in this study were obtained from the Web of Science Core Collection, and no patients or public contributions were involved in this research.

## Results

### Analysis of Publication Outputs and the Total Local Citation Score

**Figure 1:**
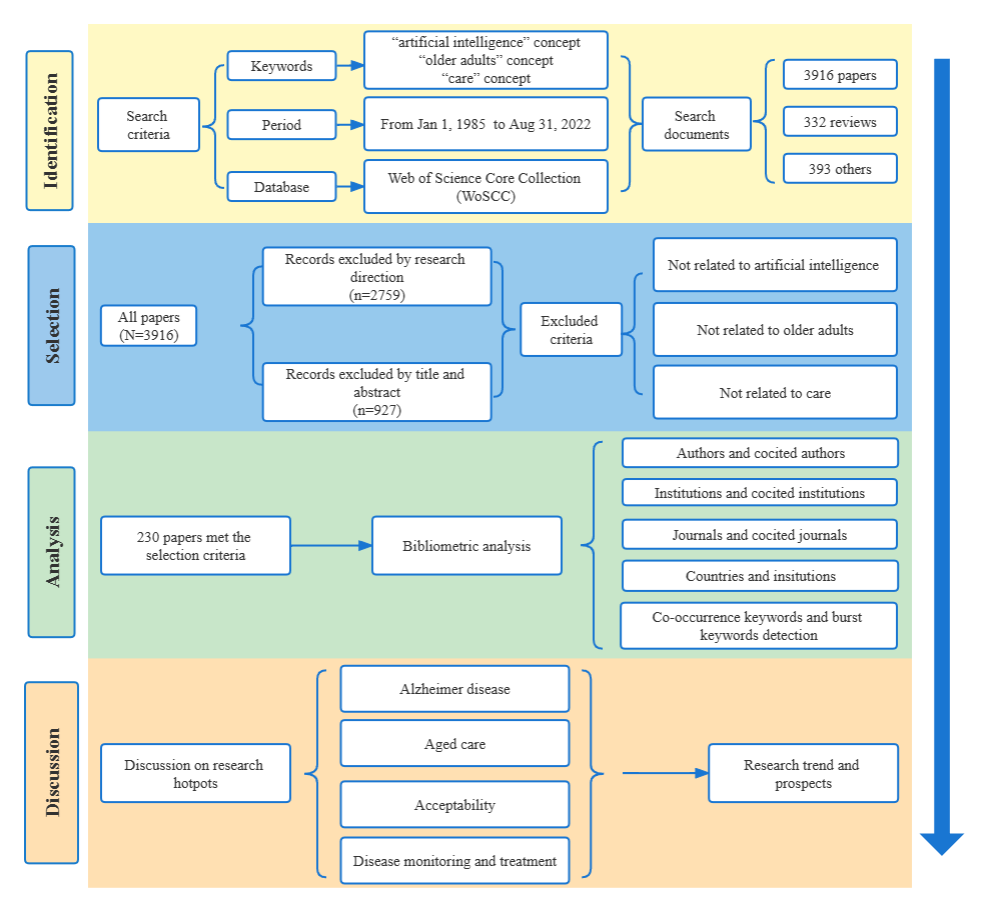
Flowchart of the literature-screening process and research framework.

**Figure 2:**
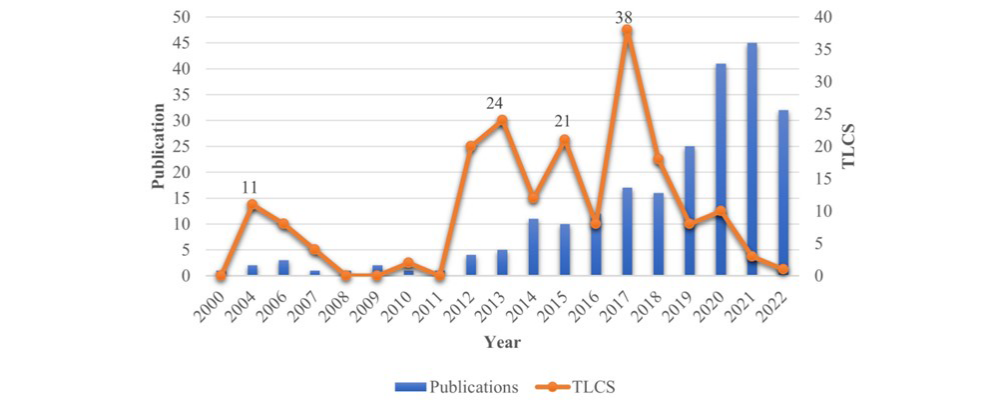
Publication output and the TLCS over time. TLCS: total local citation score.

A total of 4641 papers were searched from the Web of Science Core Collection, and 230 (4.96%) papers were included in the analysis after the data were cleaned. Figure 1 shows the literature-screening process and research framework, and the annual number of publications is shown in Figure 2. The number of publications on the application of AI in geriatric care has steadily increased, from the first paper in 2000 to 32 papers in 2022. Publications increased sharply from 2014 to 2022, accounting for 90.87% (209/230) of all publications. However, the variation in the total local citation score (TLCS) in this field is not smooth. In contrast, the later the papers are published, the lower their times of being cited. The higher the TLCS, the more important the publication in the field. The TLCS peaked 4 times during the period covered by our study (in 2004, 2013, 2015, and 2017), suggesting that these were pivotal years in which seminal literature may have been published.

Our results support the correlation between the peaks in the TLCS and the publication of seminal literature. We found that Tamura et al [21] conducted a study of 13 older adults with severe Alzheimer disease to investigate the effectiveness of entertainment robots in occupational therapy in 2004. The results showed that during occupational therapy, entertainment robots effectively increase the social activity of patients with Alzheimer disease. The entertainment robot is an effective rehabilitation tool for the treatment of patients with severe dementia. In addition, the study [21] also proposed that entertainment robots could be substituted for therapy animals in critical care units. Robots are safer than animals, but their price is another issue. Opinions differ as to whether using robots would be worthwhile. We also found that Moyle et al [22] conducted a pilot randomized controlled trial in 2013 to explore the effects of companion robots on the emotional expression of older adults with moderate-to-severe dementia in a residential care setting. The findings indicated that companion robots have a moderate-to-very-positive influence on patients quality of life, resulting in significant improvements in speech function and emotional expression and a reduction in loneliness [22]. In 2015, Sung et al [23] carried out a 1-group before-and-after study to evaluate the effects of robot-assisted therapy on older adults in many long-term care facilities. The participants’ communication, interaction skills, and activity levels significantly improved after receiving 4-week robot-assisted therapy [23]. Robot-assisted therapy could be a daily activity and has the potential to improve the social health of older adults in residential care facilities. In 2017, Hebesberger et al [24] used a mixed methods study to evaluate the social acceptance of and experiences with long-term autonomous robot use by staff and older adults in nursing facilities. This being the first study to focus on the impact of long-term robot exposure on user experience and acceptance, the results showed that older adults and staff felt ambivalent about the robot. On the one hand, they were curious about and engaged with the robot; they enjoyed its presence. On the other hand, the older adults expressed fear and rejected the use of the robot, and the staff was not willing to share their workspace with a robotic aid [24].

### Analysis of Countries/Regions and Institutions

A total of 39 countries/regions and 499 institutions had participated in the publication of the 230 papers. The top 10 countries/regions for publications are shown in Table 1. The United States had the highest number of publications during the study period (n=63, 27.39%), followed by China (n=41, 17.83%) and Japan (n=28, 12.17%). In terms of the number of papers published, the top 10 countries/regions exceeded 90.00% of all the papers, suggesting that research in this area is unevenly developed among countries/regions.

Table 2 shows the top 10 institutions in terms of publications in the field. Griffith University and the University of Auckland had the highest number of publications during the study period (n=10, 4.35%), followed by the University of Toronto (n=6, 2.61%), Bond University (n=5, 2.17%), and the University of Pennsylvania (n=5, 2.17%).

**Figure 3:**
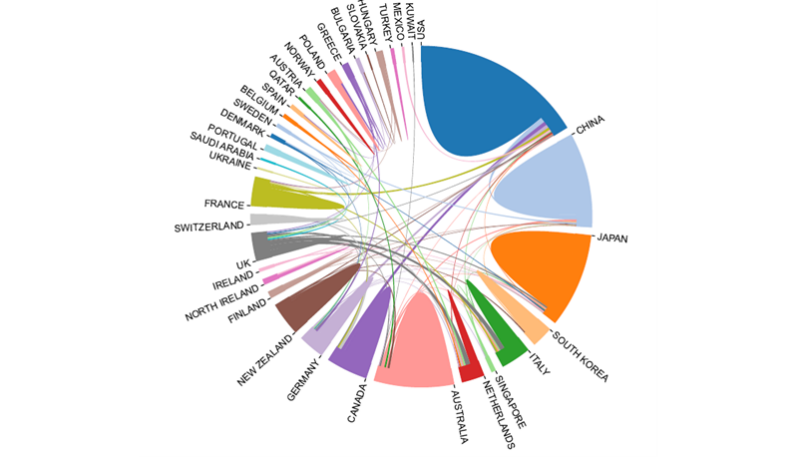
Network map of scientific cooperation among countries/regions.

Wuchty et al [25] showed that teams often publish more impactful research than individuals and that analysis of the collaborative relationships among different countries/regions, institutions, and authors can also reflect the scholarly exchange in this field. Figure 3 shows the cooperation among countries/regions. The line between countries reflects the cooperation among them, and they are mostly in cooperation with one another. It is worth noting that the more frequent the exchange among countries, the more the output, and this is the case with the United States. The figure shows that the lines crossing with the United States are numerous and thick, indicating that the United States plays an important role in the development of cooperation in this field. Thus, the United States also produces the most output and cooperates closely with China, Canada, Germany, France, and Japan.

**Table 1:** Top 10 countries/regions by number of publications.

Rank	Country	Publications, n (%)	TLCS^a^
1	United States	63 (27.39)	39
2	China	41 (17.83)	17
3	Japan	28 (12.17)	17
4	Australia	24 (10.43)	41
5	United Kingdom	23 (10.00)	36
6	Germany	19 (8.26)	21
7	Canada	14 (6.09)	18
7	Italy	14 (6.09)	4
8	France	13 (5.65)	10
8	New Zealand	13 (5.65)	21

^a^TLCS: total local citation score.

**Table 2:** Top 10 institutions by number of publications.

Rank	Institution	Publications, n (%)	TLCS^a^
1	Griffith University (Australia)	10 (4.35)	27
1	The University of Auckland (New Zealand)	10 (4.35)	21
2	University of Toronto (Canada)	6 (2.61)	6
3	Bond University (Australia)	5 (2.17)	4
3	University of Pennsylvania (United States)	5 (2.17)	2
4	Queensland University of Technology (Australia)	4 (1.74)	16
5	Angers University Hospital (France)	3 (1.30)	2
5	Columbia University (United States)	3 (1.30)	0
5	University of Heidelberg (Germany)	3 (1.30)	0
5	Maastricht University (the Netherlands)	3 (1.30)	3

^a^TLCS: total local citation score.

### Analysis of Journals and Citations

Journal analysis can help find the core journals in this field [26,27]. AI has been widely used in various applications, including games, automation, medicine, and process control [28]. Increasingly more researchers and scientists have begun to engage in this theme. The analysis revealed that 124 journals were involved in research on the application of AI in geriatric care during our study period. Table 3 shows the top 10 journals in terms of the number of publications and their respective TLCS. The *International Journal of Social Robotics* published the largest number of publications (n=22, 9.57%) on this topic. The impact factor of this journal is 3.802. It was followed closely by the *Journal of the American Medical Directors Association* (n=11, 4.78%), *Assistive Technology* (n=6, 2.61%), and the *Journal of Medical Internet Research* (n=6, 2.61%). The journals with the highest TLCS were the *International Journal of Social Robotics* (TLCS=27) and the *Journal of the American Medical Directors Association* (TLCS=32), indicating that these 2 journals are likely to be the definitive publications for the application of AI in geriatric care.

**Table 3:** Top 10 journals that published papers on the application of AI^a^ in geriatric care.

Rank	Journal	Publications, n (%)	TLCS^b^	IF^c^
1	*International Journal of Social Robotics*	22 (9.57)	27	3.802
2	*Journal of the American Medical Directors Association*	11(4.78)	32	7.802
3	*Assistive Technology*	6 (2.61)	5	2.170
3	*Journal of Medical Internet Research*	6 (2.61)	0	7.077
4	*Journal of Alzheimers Disease*	5 (2.17)	17	4.160
5	*Advanced Robotics*	4 (1.74)	2	2.057
5	*BMC Geriatrics*	4 (1.74)	0	4.070
5	*Frontiers in Aging Neuroscience*	4 (1.74)	0	5.702
5	*Journal of Clinical Nursing*	4 (1.74)	1	4.423
5	*Journal of Gerontological Nursing*	4 (1.74)	17	1.436

^a^AI: artificial intelligence.

^b^TLCS: total local citation score.

^c^IF: impact factor (Journal Citation Reports 2021).

### Analysis of Publications and Citations

To some extent, the citation ranking of a paper can also explain the research hotspots in the academic field [29]. The title of a paper states the paper’s subject. Based on the TLCS, Table 4 lists the top 10 most frequently cited publications. Of the top 10 papers, 7 (70%) focused on the impact of robotics on people with Alzheimer disease, indicating that the application of AI to patients with dementia is the primary research question.

**Table 4:** Top 10 papers by the TLCS^a^.

Rank	Title (author)	Year	Journal	TLCS
1	Exploring the Effect of Companion Robots on Emotional Expression in Older Adults With Dementia: A Pilot Randomized Controlled Trial (Moyle et al [22])	2013	*Journal of Gerontological Nursing*	17
2	The Utilization of Robotic Pets in Dementia Care (Petersen et al [30])	2011	*Journal of Alzhimers Disease*	16
3	Effects on Symptoms of Agitation and Depression in Persons With Dementia Participating in Robot-Assisted Activity: A Cluster-Randomized Controlled Trial (Joranson et al [31])	2015	*Journal of the American Medical Directors Association*	12
4	Is an Entertainment Robot Useful in the Care of Elderly People With Severe Dementia? (Tamura et al [21])	2004	*Journal of Gerontology Series A-biological Sciences and Medical Sciences*	11
5	Attitudes Towards Health-Care Robots in a Retirement Village (Broadbent et al [32])	2012	*American Journal of on Ageing*	10
5	Use of a Robotic Seal as a Therapeutic Tool to Improve Dementia Symptoms: A Cluster-Randomized Controlled Trial (Moyle et al [33])	2017	*Journal of the American Medical Directors Association*	10
6	In the Hands of Machines? The Future of Aged Care (Sparrow and Sparrow [34])	2006	*Minds and Machines*	8
7	Care Staff Perceptions of a Social Robot Called Paro and a Look-Alike Plush Toy: A Descriptive Qualitative Approach (Moyle et al [35])	2018	*Aging & Mental Health*	7
8	Social Commitment Robots and Dementia (Roger et al [36])	2012	*Canadian Journal on Aging-Revue Canadienne Du Vieillissement*	6
9	Robot-Assisted Therapy for Improving Social Interactions and Activity Participation Among Institutionalized Older Adults: A Pilot Study (Sung et al [23])	2015	*Asia-Pacific Psychiatry*	5

^a^TLCS: total local citation score.

### Analysis of Authors and Coauthorship Networks

**Figure 4:**
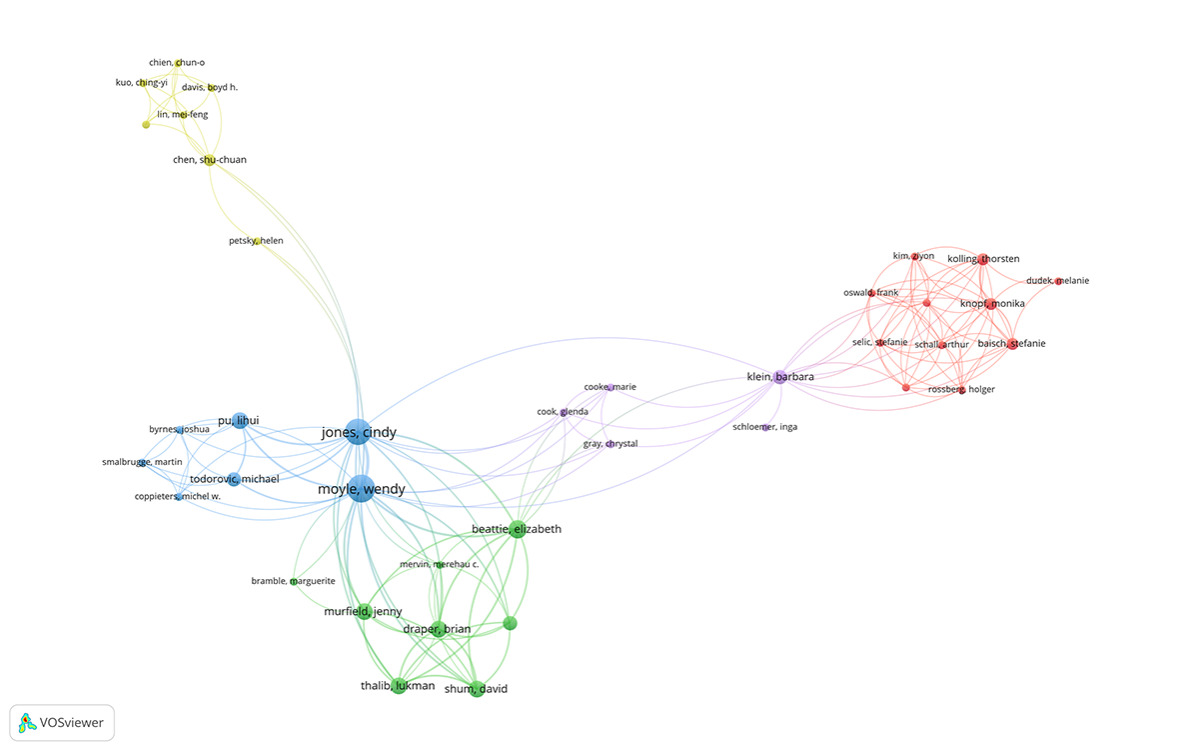
Visualization of research networks of authors with a minimum of 1 paper [38].

The high citation count (h-index) was proposed by Jorge E Hirsch of the University of California, San Diego, USA, in 2005. It is a mixed quantitative metric used to assess the scholarly achievement of researchers. The higher the h-index, the greater the academic impact. The h-index indicates that a person has “h” papers, each of which has been cited at least “h” times in a given period [37]. In total, 977 authors took part in related studies in the past. Table 5 presents the top 10 authors in terms of the number of published papers. The top 3 (30%) are Wendy Moyle (Australia), Cindy Jones (Australia), and Elizabeth Broadbent (New Zealand). Moyle has the most publications, and Ngaire has the highest h-index (h-index=46), which means that they are extremely influential in their field. Figure 4 illustrates the collaborative relationships among authors in this field. The different colors stand for different clusters of authors. The analysis of collaboration among all authors showed that only 38 (3.13%) authors formed a collaborative network and were divided into 5 main clusters. The thickness of lines indicates the strength of the relationship between authors relative to others. The largest cluster (in red) involved 11 (28.9%) coauthors, centering on Baishch Stefanice, Knopf Oinks, and Kolling Thorsten. The second cluster (in blue) was dominated by Moyle and Jones. They had the largest nodes and were the most active coauthors in the field. The smallest cluster (in purple) consisted of only 5 authors, but they collaborated with the largest cluster and the authors with the highest number of publications through Barbara Klein. Meanwhile, there was a lack of collaboration among the other top 10 authors involved in research in this field of publication.

**Table 5:** Top 10 authors who published research papers.

Rank	Author	Publications, n (%)	TLCS^a^	TGCS^b^	h-Index
1	Moyle (Australia)	11 (4.78)	44	436	26
2	Jones (Australia)	10 (4.35)	34	333	20
3	Broadbent (New Zealand)	9 (3.91)	21	361	40
4	Kerse (New Zealand)	5 (2.17)	12	238	46
4	MacDonald (New Zealand)	5 (2.17)	18	272	26
5	Beattie (Australia)	4 (1.74)	23	228	28
5	Johnson (United States)	4 (1.74)	2	112	16
5	Pu (China)	4 (1.74)	4	43	7
5	Thalib (Istanbul)	4 (1.74)	16	186	37
6	Muramatsu (New Zealand)	3 (1.30)	1	53	12

^a^TLCS: total local citation score.

^b^TGCS: total global citation score.

### Co-occurrence of Keywords and Burst Keyword Detection

**Figure 5:**
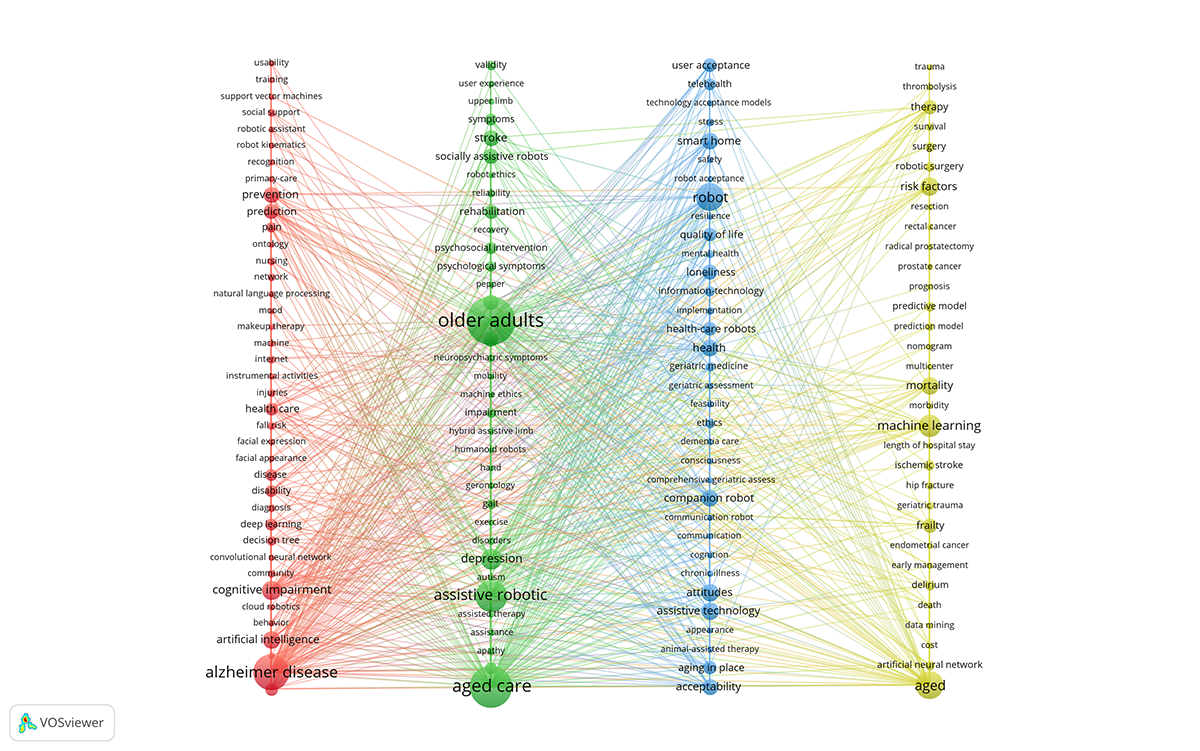
Keyword co-occurrence network [38].

Keywords are effective in analyzing the knowledge structure of an academic field from a bibliometric perspective, which can help identify potential research hotspots [39]. Thus, the themes covered in this study could be identified using the keywords. We analyzed the keywords that appeared more than 2 times. This was calculated using a clustering algorithm similar to modularity-based clustering. The titles and abstracts of 230 papers were included in the analysis; we extracted the keywords with the top 140 occurrences and presented them visually. As seen in Figure 5, the colors of the elements stand for the clusters to which they belong and the different clusters are represented by assigned colors. The node size indicates the occurrence of the keyword, and the thickness of the link represents the co-occurrence intensity. The thicker the link between nodes, the greater the co-occurrence between keywords. We clearly divided the keywords into 4 categories using 4 colors (red, green, blue, and yellow) to indicate that these topics demonstrate the mainstream research hotspots and frontier areas.

Cluster 1 is associated with Alzheimer disease. The primary keywords were “cognitive impairment,” “health care,” “prevention,” “prediction,” and “artificial intelligence.” This cluster explores the application of AI in Alzheimer disease. Cluster 2 is primarily related to aged care. The main keywords were “assistive robotic,” “rehabilitation,” “stroke,” and “socially assistive robots.” The cluster focuses on the application of AI in geriatric care. Cluster 3 is related to the acceptance of AI applications. The keywords were “user acceptance,” “robot acceptance,” and “technology acceptance models.” The cluster explores the acceptance of the applications of AI in geriatric care. Cluster 4 is related to the surveillance and treatment of diseases in older adults. The essential keywords were “machine learning,” “therapy,” “robotic surgery,” “frailty,” and “risk factors.” This cluster explores the application of AI in the monitoring and treatment of diseases in older adults. These 4 themes constitute the mainstream academic literature on the application of AI in geriatric care.

**Figure 6:**
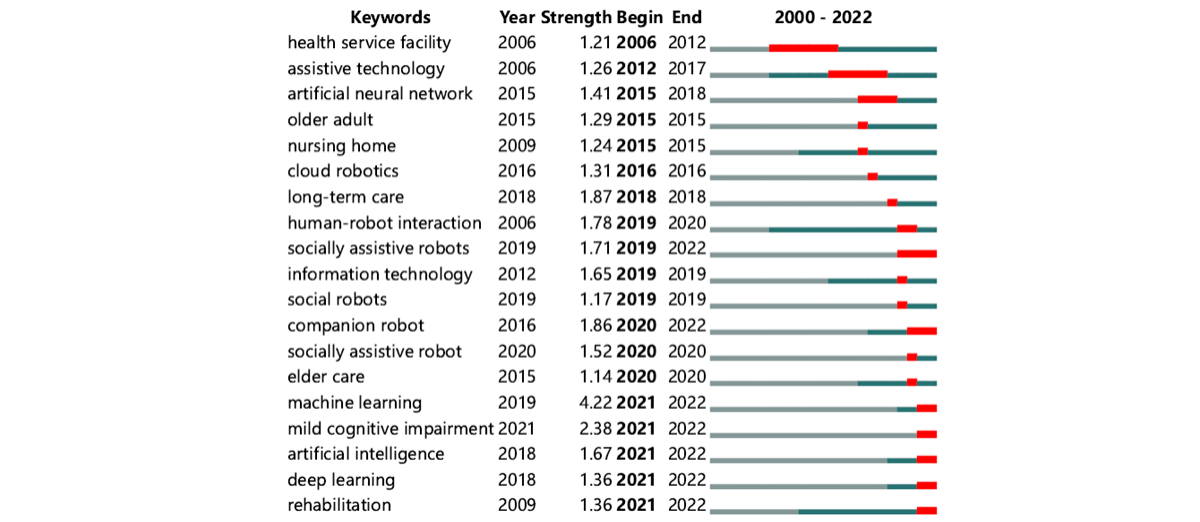
Top 19 keywords with strong citation bursts between 2000 and 2022.

Keywords that appear suddenly and are cited extensively, or relatively so, for a short period are known as burst keywords [20]. They are found using the default Kleinberg algorithm of CiteSpace. Considered important indicators of frontier research hotspots, burst keywords herald emerging trends. Figure 6 shows the top 19 burst keywords in this field for 2000-2022. The thick red line shows the period of the keyword’s outbreak. Machine learning (4.22) had the highest burst strength, followed by mild cognitive impairment (2.38). In addition, “health service facility” was the keyword with the longest burst (6 years). Recent burst keywords included “machine learning,” “deep learning,” and “rehabilitation.” These burst keywords also reflect the research trends in the field and may become a research hotspot in the future.

## Discussion

### Principal Findings

Our study shows that research on the application of AI in geriatric care has gradually increased over the past 23 years. Nevertheless, the field has developed relatively slowly and still has a lot of research space. Moreover, the development of research has been uneven among countries/regions, and cooperation among the countries/regions and institutions in which the field has been less developed is more limited. This may be related to the country’s degree of economic development and the size of its aging population. According to the 2019 global ranking of countries with aging populations, Japan, the United Kingdom, Australia, the United States, and China all rank at the top [40]. In addition, most are high-income countries. Meanwhile, the United States has the highest number of publications, which may be related to its state support. On February 11, 2019, the United States issued the executive order “Maintaining American Leadership in Artificial Intelligence,” directing all federal government agencies to implement strategic objectives aimed at accelerating AI research and development [41].

In this study, we hoped to identify research hotspots and gaps by providing a comprehensive analysis and structured information about the field. Through the analysis of highly cited studies in publications, we found that the application of robotics in the care of patients with Alzheimer disease is the primary focus of the field, and this was validated by the cluster analysis of the keywords.

Alzheimer disease is a progressive degenerative disease of the nervous system, with an insidious onset. It is a clinical syndrome, characterized by memory impairment, aphasia, agnosia, executive dysfunction,personality, and behavioral changes, etc [42]. Studies have reported that the number of people with Alzheimer disease worldwide will increase from 57 million in 2015 to 152 million in 2050, with a significant increase in its prevalence among older adults [43,44]. Many countries are facing a shortage of caregivers for patients with Alzheimer disease [45]. With the development of information technology, many studies have focused on the application of AI to the treatment of older people with Alzheimer disease. For example, companion robots improve the quality of life, mood, and cognitive abilities of patients with Alzheimer disease [46,47]. Several studies have demonstrated the effectiveness and feasibility of AI in Alzheimer care [48]. However, various factors, including cost and environmental constraints, limit the use of AI in the treatment of patients with Alzheimer disease [49]. Promoting the use of AI in the treatment of patients with Alzheimer disease may be a direction for future research.

The application of AI in geriatric care is another research hotspot in this area. As aging continues to deepen, geriatric care has become a social issue in urgent need of being solved. Currently, AI is used widely in the daily care of older people. AI can assist older adults with their daily tasks, such as eating, bathing, and dressing [50-52]. It can also help rehabilitate older adults with disabilities, improving their quality of life and increasing their independence [53-56]. Concurrently, many other researchers have explored socially assistive robots. For example, these robots are said to be promising in terms of alleviating feelings of loneliness and social isolation [57-59]. However, there are still 2 problems that we should think about: first, how we can guarantee the safety of AI in the home environment, and second, how older adults face the “digital divide” brought about by AI.

In recent years, the acceptance of technology has become a hotspot. Needless to say, the population’s acceptance of technology affects the application and development of AI. If the target user is resistant to AI, they will be immune to its advantages. Hence, several recent studies have focused on people’s acceptance of AI. Some scholars have investigated the acceptable behavior and influencing factors of older people for smart geriatric care services, companion robots, daily care robots, and social assistance robots for care and companionship in daily life [60-65]. One study examined the acceptance of home care robots by older people in Finland, Ireland, and Japan. Intriguingly, the results showed that each country has a different view. More older people in Finland have a negative impression of robots compared to their peers in the other 2 countries [66]. These differences may be related to the history, culture, policies, and values associated with the development of AI in each country. We give full consideration to the actual situation of each country throughout the process of promoting the application of AI in the future. However, there are fewer studies on how to increase people’s acceptance of AI, which may be a direction for future research.

Through an analysis of the literature, we found that AI plays a vital role in monitoring the diseases and treatment of older people. The declining physiological function in older people leads to a significant increase in their morbidity and disability. This phenomenon has led to an increasing demand for medical services by older people [4]. AI can help health care workers not only monitor but also treat the medical conditions of older people. Furthermore, academics have paid a lot of attention to this research hotspot. Health care professionals can monitor the status of older people through telemedicine, for example, wearable smart devices and robots, which can help them assess and improve their patients’ conditions remotely and dynamically [67,68]. In recent years, several researchers have studied the application of machine learning, which is mostly used in the diagnosis and management of diseases. Examples include screening populations with mild cognitive impairment (MCI) or early Alzheimer disease and predicting the incidence of delirium risk after patients’ hip fracture surgeries [69,70]. Future research could try to use machine learning to monitor other disease conditions in older people, and it might be a research direction worth pursuing.

### Limitations

Inevitably, we need to acknowledge the limitations of this study. First, due to the applicability of the software, the study only searched the Web of Science database, which is the most influential multidisciplinary academic literature abstract index database worldwide. Holding more than 12,400 authoritative and high-impact academic journals worldwide, the core collection of this database has become an important tool for academic analysis and evaluation. However, because we limited our search to just 1 database, we may have missed some important research results [71]. Additionally, our keyword-cleaning process and statistics are self-designed and may be limited by our professional knowledge and experience. In the future, we will further expand our data sources and standardize keywords to help us enhance the overall quality of the paper and the accuracy of our forecasts.

### Conclusion

In this bibliometric analysis study, we delineated the trajectory of research on AI in geriatric care in the past 23 years. Nevertheless, the development of research and cooperation among countries/regions and institutions are limited in a number of instances. Study suggests that researchers should focus on interinstitutional collaborations, especially international cooperation, to advance AI research [72]. Therefore, strengthening the cooperation and communication between different countries/regions and institutions will contribute to the further development of this field. At present, the field is focused on the care of older adults, the surveillance and treatment of their diseases, the acceptance of AI applications, and Alzheimer disease. This study provides researchers with the information necessary to understand the current state, collaborative networks, and main research hotspots in the field. At the same time, our results suggest a series of recommendations for future research.

## Abbreviations

AI: artificial intelligence
TLCS: total local citation score

## Data Availability

The data sets generated and analyzed during this study are available from the corresponding author upon reasonable request.
